# Evaluation of Naringenin as a Promising Treatment Option for COPD Based on Literature Review and Network Pharmacology

**DOI:** 10.3390/biom10121644

**Published:** 2020-12-08

**Authors:** Zhen Chen, Pan Chen, Hao Wu, Rui Shi, Weiwei Su, Yonggang Wang, Peibo Li

**Affiliations:** Guangdong Engineering and Technology Research Center for Quality and Efficacy Re-Evaluation of Post-Marketed TCM, State Key Laboratory of Biocontrol and Guangdong Provincial Key Laboratory of Plant Resources, School of Life Sciences, Sun Yat-sen University, Guangzhou 510275, China; chenzh358@mail2.sysu.edu.cn (Z.C.); chenpan989@126.com (P.C.); wuhao_cpu@126.com (H.W.); ruishi900930@gmail.com (R.S.); lsssww@mail.sysu.edu.cn (W.S.); wangyg@mail.sysu.edu.cn (Y.W.)

**Keywords:** COPD, naringenin, pathogenesis, mechanism

## Abstract

Chronic obstructive pulmonary disease (COPD) is a chronic respiratory disease characterized by incompletely reversible airflow limitation and seriously threatens the health of humans due to its high morbidity and mortality. Naringenin, as a natural flavanone, has shown various potential pharmacological activities against multiple pathological stages of COPD, but available studies are scattered and unsystematic. Thus, we combined literature review with network pharmacology analysis to evaluate the potential therapeutic effects of naringenin on COPD and predict its underlying mechanisms, expecting to provide a promising tactic for clinical treatment of COPD.

## 1. Introduction

### 1.1. Chronic Obstructive Pulmonary Disease

Chronic obstructive pulmonary disease (COPD), one of the most common chronic respiratory disease, is characterized by progressive and irreversible airflow limitation resulting from the emphysematous destruction of the alveolar structure and the remodeling and narrowing of small airways [1,2]. COPD is considered as a multifactor disease, and cigarette smoking is demonstrated as the dominant driving force for the development of the disease [3]. Since its high prevalence, morbidity, and mortality, COPD induces substantial economic and social burden worldwide. It is predicted that COPD will become the third-ranked leading disease of death worldwide in 2030, and there may be over 5.4 million deaths annually from it in 2060 due to the increasing numbers of smokers and aging populations [4,5].

Clinical phenotypes of COPD vary among patients due to the differences in the age of onset, the rate of progression, the frequency of exacerbations, and the association with comorbidities, with some patients predominantly suffering from small airway disease, while others mainly suffer from pulmonary diseases such as emphysema [6]. Although several treatments of COPD, including inhaled corticosteroids, long-acting muscarinic antagonists, and long-acting β2-agonists have already demonstrated to have a certain degree of clinical efficacy, it seems that the side effects of these currently available therapies are unavoidable and time- or dose-dependent [7]. In addition, the precise mechanisms of COPD pathogenesis have not been clarified at present. Therefore, it is critical to elucidate the molecular mechanisms underlying COPD and identify an alternative ingredient that can treat COPD with fewer side effects.

### 1.2. Naringenin and its Glycoside Naringin

**Figure 1:**
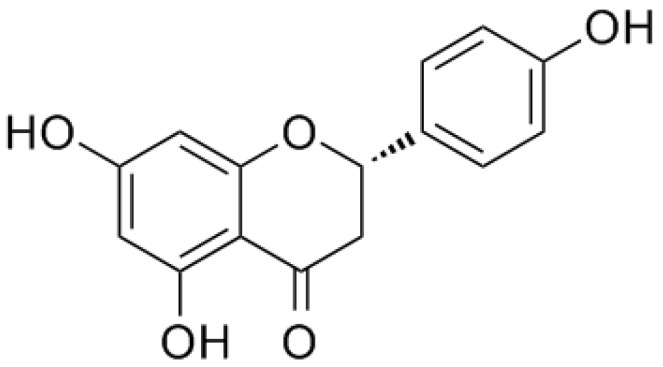
Chemical structure of naringenin.

Naringenin, a natural flavanone, was first identified from extracts of the dormant peach (*Prunus persica*) flower buds, with the chemical name of 5,7,4′-trihydroxyflavanone [8] (Figure 1). As a common dietary constituent consumed by humans, naringenin is abundantly present in citrus fruits and vegetables such as grapefruit, lemon, oranges, and tomatoes. Naringin is a flavanone glycoside composed of naringenin and neohesperidose attached at C-7, which is partly absorbed by gastrointestinal tracts and is mostly metabolized by gastrointestinal bacteria into naringenin after oral ingestion [9]. Thus, naringin is mainly introduced into the body as a form of naringenin [10]. In recent years, accumulating studies have reported on the potential pharmacological activities of naringenin, including beneficial effects in chronic airway disease, lung diseases, liver diseases, cardiovascular diseases, and cancer [11,12,13,14,15]. Evidence suggests that it had antioxidative, anti-inflammatory, antifibrogenic, antiatherogenic, and antiproliferative bioactivities [16,17,18]. Even though its therapeutic effects in the treatment of COPD are seldom reported, these findings still indicate that naringenin and its glycoside naringin appear to be full of potential therapeutic value in COPD.

The traditional concept of “one drug for one target for one disease” was the predominant paradigm in drug discovery in the past. However, advances in systems biology suggest that complex diseases may not be effectively treatable by interventions at single targets [19]. As a classic method of bioinformatics, network pharmacology can save cost and time compared with conventional experiments, and, more importantly, the core of network pharmacology is consistent with the holistic philosophy, which contributes to overcoming complex diseases such as COPD, in a systematic manner [20,21]. Consequently, in this study, we combined literature review with network pharmacology analysis to evaluate the possible therapeutic effect of naringenin on COPD and its underlying mechanisms, expecting to provide a promising treatment option for COPD.

In conclusion, naringenin exhibits various pharmacological effects against multiple respiratory diseases, which suggests its potential therapeutic effect on different pathological stages of COPD (Table 1).

## 2. Potential Pharmacological Effects of Naringenin in COPD

### 2.1. Anti-Inflammatory Activity

Accumulating reports suggested that persistent inflammation in the lung parenchyma and peripheral airways plays a critical role in the initiation and progression of COPD [22]. The pathogen- and damage-associated molecular patterns initiate the immune response at the early phase of this inflammation. Pro-inflammatory cytokines, chemokines, and activated inflammatory cells are generated, resulting in chronic bronchitis or emphysema. In multiple animal models, orally administered naringenin or naringin was proved to markedly reduce the infiltration of inflammatory cells and decrease the myeloperoxidase activity in the lungs, thereby exerting protective effects to ameliorate the histopathological lung tissue injury resulting from lipopolysaccharide (LPS), CS exposure, *Staphylococcus aureus*, or cecum ligation and puncture (CLP) [23,24,25,26].

Clinical trials revealed that the elevated levels of pro-inflammatory cytokines such as tumor necrosis factor (TNF)-α, interleukin (IL)-8, IL-6, and IL-1β in the serum and sputum of COPD patients, which is associated with the severity and exacerbation frequency of this disease [27,28,29]. Specific pro-inflammatory cytokine such as IL-8 and IL-1β blockade therapies are currently emerging and have demonstrated as a certain degree of efficacy in COPD [30,31,32]. Liu et al. reported that naringenin could attenuate inflammation in CS-exposed mice and involve the suppression of NF-κB [33]. Naringenin (20, 40, and 80 mg/kg, p.o.) could inhibit the production of IL-8 and TNF-α and decrease the level of matrix metalloproteinase (MMP)-9 in the bronchoalveolar lavage fluid (BALF) and serum. Similarly, inhibition of TNF-α and IL-8 by treatment of naringin (9.2, 18.4, and 36.8 mg/kg, p.o.) was observed in the airways of guinea pigs with chronic bronchitis challenged by CS exposure [34]. IL-1β and IL-6 often synergistically work with other cytokines and thus thereby provide a link between innate and acquired immunity in COPD [35]. Zhao et al. found that naringenin (100 mg/kg, p.o.) pre-treatment could significantly decrease the serum and BALF levels of IL-1β and IL-6 in an LPS-induced acute lung injury mouse model, which is probably correlated with suppressing the phosphatidylinositol 3-kinase (PI3K) and protein kinase B (AKT) signaling pathways [36]. Zhang et al. reported that orally administrated naringenin (100 and 200 mg/kg, p.o.) could ameliorate lung injury by downregulating the level of IL-1β in radiation-induced mice [37]. Moreover, naringenin (50 and 100 mg/kg, p.o.) was observed to decrease the level of IL-6 in lung tissues, thereby attenuating LPS-induced acute lung injury in rats [12]. An opponent of Th1 cells, Th2 cells are characterized by the production of anti-inflammatory cytokines such as IL-4, IL-5, IL-10, and IL-13, which were reported to be reduced in COPD patients [38,39,40,41,42]. Recently, blocking antibodies against Th2 cytokines and their receptors have shown clinical benefits in COPD and asthma [43,44,45]. Ahmad and colleagues investigated the anti-inflammatory mechanisms of naringin against carrageenan-induced pleurisy in a mouse model [46]. Orally administered naringin (40 and 80 mg/kg) was shown to downregulate Th1 cytokines (TNF-α, IL-2, IL-6, and IL-17) and upregulate Th2 cytokines (IL-4 and IL-10) in the pleural exudates, through the inhibition of NF-κB and STAT3 signaling pathways. In a CS-challenged rat model, naringin (20, 40, and 80 mg/kg, p.o.) was found to dose-dependently elevate the level of IL-10 in BALF [24].

Chemokines are mainly divided into four subfamilies including CC chemokines, CXC chemokines, XC chemokines, and CX3C chemokines, which play a critical role in recruiting inflammatory cells from the circulation into the lungs in COPD [47,48]. Liu et al. evaluated the effect of naringin on chemokine expression in LPS-challenged RAW 264.7 macrophages [49]. Pre-treatment with naringin (50, 100, and 200 μM) was found to significantly reduce the secretion of monocyte chemoattractant protein (MCP)-1 and macrophage inflammatory protein (MIP)-1α. Shi et al. investigated the anti-inflammatory mechanism of naringenin in an allergen-induced murine model of asthma. Naringenin (25, 50, and 100 mg/kg, i.p.) was observed to markedly reduce the levels of CCL5 and CCL11 in the BALF, which is correlated with blocking the activation of NF-κB [50]. Thymic stromal lymphopoietin (TSLP), an IL-7-like cytokine, can synergize with IL-1β and TNF-α to induce Th2-differentiated cytokines and chemokines expression in mast cells [51]. A clinical trial showed that the expression of TSLP, CCL17, CCL22, and CXCL10 increased in the bronchial mucosa and BALF of COPD patients [52]. Naringenin (100 μM) was found to inhibit TSLP production at a maximal rate of 62.27 ± 10.79% probably through suppressing the receptor-interacting protein (RIP)-2 and caspase-1 in human mast cell line [53].

Owing to the hydrophobic structure of naringenin, it possesses a poor aqueous solubility and bioavailability. As a drug delivery system, naringenin nanocarriers are currently emerging so that promote the bioavailability and enhance the therapeutic effect of naringenin [54]. Kumar et al. investigated the anti-inflammatory mechanisms of a novel naringenin delivery system in LPS-induced RAW264.7 macrophage cells [55]. Compared with naringenin, polyvinyl pyrrolidone (PVP) coated-naringenin nanoparticles (NPs) were shown to be more efficient. These naringenin NPs (25 μg/mL) were observed to downregulate the expression of NF-κB via the P38 mitogen-activated protein kinase (MAPK) signaling pathway and to inhibit the production of inflammatory mediators including TNF-α, IL-6, MCP-1, and IL-1β. The anti-inflammatory activities of naringenin and naringin are summarized in Table 1.

### 2.2. Antioxidative Activity

Oxidative stress is considered as another driving mechanism in COPD pathogenesis [56]. It is suggested that reducing oxidative stress by antioxidants or enhancing endogenous antioxidant capacity may be feasible therapeutic tactics for COPD. N-acetylcysteine is a potential add-on therapy in COPD because of its antioxidant properties, but its clinical management in the treatment of COPD has remained controversial primarily due to its reduced bioavailability in oral form and its acidic nature debarring its use as an inhaled form [57]. Barnes considered that there are currently no safe and effective antioxidants for the treatment of COPD, possibly because of the difficulties of identifying which patients would benefit most from antioxidant therapy and the dose needed to restore the redox balance in COPD patients [58].

Multiple studies have shown that, compared with healthy controls, hydrogen peroxide (H_2_O_2_) was greatly increased in the exhaled breath condensate of COPD patients, which is correlated with forced expiratory volume in one second, neutrophil count, and dyspnea score [59,60,61]. Naringenin (100 mg/kg, p.o.) was observed to downregulate the levels of reactive oxygen species (ROS) including H_2_O_2_ and malondialdehyde (MDA) in the BALF of LPS-challenged acute lung injure mice, suggesting its potential antioxidative activity in pulmonary diseases [36]. The activities of common enzymatic antioxidants including superoxide dismutases (SOD), catalases (CAT), and glutathione peroxidases (GPx) were found to be significantly increased in patients with COPD compared to healthy controls subjects and they are proportionate to the severity of the disease [62,63]. In addition, the cyclooxygenase (COX)-2 and nuclear factor erythroid-2 related factor (Nrf)2 mediating signaling pathway has been considered as a new approach for preventing oxidative stress and inflammation in COPD [64,65]. Ali et al. investigated the antioxidative mechanisms of naringenin in a benzo[a]pyrene-induced Wistar rat model [66]. Pre- or post-treatment with naringenin (100 mg/kg, p.o.) was found to not only significantly enhance the levels of glutathione (GSH) and GSH-dependent enzymes such as GPx, glutathione s-transferase (GST), and glutathione reductase (GR), but also increase the levels of SOD, CAT, and xanthine oxidase (XO) both in BALF and in lung tissues. Further immunohistochemical analyses revealed that naringenin could also suppress the expression of COX-2 through blocking the activation of NF-κB in lung tissues. Podder et al. accessed the cytoprotective effect of naringenin against paraquat-induced cellular toxicity in the human bronchial epithelial BEAS-2B cell line [67]. Naringenin (100 μM) was observed to decrease the generation of ROS and induce the expression of antioxidant-related genes including GPX2, GPX3, GPX5, and GPX7. Further study revealed that naringenin exerts anti-oxidative activity probably associated with the activation of the Nrf2 signaling pathway.

Nitric oxide (NO) may be generated by type 2 nitric oxide synthase (NOS) (also known as inducible NOS, or iNOS), which was significantly increased in patients with COPD compared with non-smokers and smokers with normal lung function [68,69]. Akintunde et al. reported the potential antioxidative activity of naringin in a wood smoke exposure-induced rat model [70]. Naringin (80 mg/kg, p.o.) was shown to not only increase the activities of SOD and CAT but also lower the levels of NO in the lung tissues, thereby ameliorating the pulmonary damage. Naringenin (50 mg/kg, p.o.) was also found to significantly increase GSH content and endothelial nitric oxide synthase (eNOS) protein expression, whereas decreased the expression of iNOS in both lung and heart tissues in monocrotaline-induced pulmonary hypertension rats [71]. In addition, PVP coated-naringenin NPs were shown to suppress the expression of iNOS and COX-2 and inhibit the production of NO [55]. The antioxidative activities of naringenin and naringin are summarized in Table 2.

### 2.3. Anti-Airway Remodeling Activity

Airway remodeling is a direct cause of airflow limitation in COPD patients with hyperplasia of the airway epithelial cells, thickening of the reticular basement membrane, airway smooth muscle proliferation, deposition of collagen, and airway fibrosis [72]. The currently available therapies for airway remodeling in COPD are mainly bronchodilators and glucocorticosteroids, but patients are poorly controlled by them [73]. Although the reversibility of airway remodeling was observed in animal models, there is no available therapy proven to reverse airway remodeling in patients with COPD or asthma [74,75].

In a house dust mite-induced asthma mouse model, Seyedrezazadeh et al. revealed that the combination of hesperetin (7 mg/mL, p.o.) and naringenin (9 mg/mL, p.o.) could significantly decrease subepithelial fibrosis, smooth muscle hypertrophy in airways and lung atelectasis [76]. Both in vivo and in vitro studies have shown that airway remodeling can cause the increased expression of Th2 cytokines, which are often triggered by allergens in asthma [77,78]. Allergens induce the production of immunoglobulin E (IgE), thereby resulting in airway remodeling [79]. Xiong et al. reported that the anti-asthmatic effects of naringin in a mouse model challenged by ovalbumin [80]. Naringin (5 and 10 mg/kg, p.o.) was found to reduce mean airway resistance measured by the forced oscillation technique and the level of IgE in serum and BALF. Flow cytometric analysis revealed that the percentage of Th1/Th2 cells in naringin treatment groups was significantly higher than those in the model group. Shi et al. investigated the effects of naringenin in another ovalbumin-induced asthma mouse model [81]. Naringenin (50 mg/kg, i.p.) was found to significantly reduce the area of airway fibrosis in airways and the levels of Th2 cytokines in the BALF, thereby delaying the progression of airway remodeling. In addition, naringin (20, 40, and 80 mg/kg, p.o.) was also observed to dose-dependently reduce the thickening of the bronchial wall in CS-exposed rats [24]. The anti-airway remodeling activities of naringenin and naringin are summarized in Table 3.

### 2.4. Anti-Pulmonary Fibrosis Activity

Pulmonary fibrosis, one of the most common comorbidities that accompany COPD, is characterized by fibroblast proliferation, ECM aggregation, inflammatory damage, and structural destruction in the lungs [82,83]. A study reported by Divo et al. showed that the risk of death of COPD patients was closely associated with coexistent comorbid conditions such as pulmonary fibrosis [84]. As a result, a better understanding of the therapeutic tactics linking COPD with pulmonary fibrosis might assist in improving clinical outcomes.

The overexpression of tissue inhibitor of metalloproteinase (TIMP)-1 activates fibroblasts and thereby initiates fibrosis by inhibiting MMP-mediated ECM degradation, resulting in emphysema involved in COPD [85]. Naringin (60 and 120 mg/kg, p.o.) was found to significantly downregulate the expression of TNF-α, MMP-9, and TIMP-1 in a paraquat-induced pulmonary fibrosis mouse model. Meanwhile, the reduction of pulmonary fibrosis deposition was also observed [86]. Hydroxyproline (HYP) plays a crucial role in the pathogenesis of diseases associated with dynamically balanced collagen synthesis and catabolism such as idiopathic pulmonary fibrosis [87]. Turgut et al. found that oral treatment with naringin (80 mg/kg, p.o.) could markedly reduce the levels of HYP and lung collagen content, thereby exerting protective effects against bleomycin-induced fibrosis in Wistar rats [88].

The severity of COPD is probably correlated with transforming growth factor (TGF)-β signaling pathway-mediated polymorphisms [89]. Lin et al. reported that naringenin (100 mg/kg, p.o.) could suppress the level of TGF-β in mice serum and markedly inhibit the expression of proteins associated with fibrosis including alpha-smooth muscle actin (α-SMA), collagen I, and collagen III in BEAS-2B cells. Meanwhile, autophagy inhibition could reverse *Mycoplasma pneumoniae*-induced pulmonary fibrosis-related protein expression, suggesting that autophagy progression might play a critical role in the inhibition of pulmonary fibrosis [90]. The anti-pulmonary fibrosis activities of naringenin and naringin are summarized in Table 4.

### 2.5. Expectorant

In COPD, the mucus layer is vulnerable to destruction, thus leading to detrimental effects on lung function and homeostasis. Mucus hypersecretion may also result in airway obstruction as mucus occupies the airway lumen and inclines to be retained due to ciliary dysfunction [91,92]. Accumulating evidences have been revealed the tremendous complexity of the expression, interactions, and functions of mucins in patients with different severity of COPD, suggesting that modulating the synthesis, secretion, or structure of mucins in these patients might be a useful treatment for this disease [93]. Several natural compounds such as flavonoids have shown their potential effects on the expression and secretion of mucin [94].

Lin et al. reported the expectorant activity of naringenin in several animal models [95]. Naringenin (30–67 mg/kg, p.o.), by measuring the tracheal output of phenol red, was found to significantly increase the volume of airway secretions in mice. In unanesthetized pigeons, naringenin (90 mg/kg, p.o.) dose-dependently facilitated the mucociliary clearability and increased the tracheal mucociliary velocity 1.44-fold compared to the control by using a migration method of carbon granules. Meanwhile, treatment with naringenin (100 μM) was observed to enhance the basal lysozyme secretion from the rat tracheal ring explants, and to inhibit the LPS-induced increased mucin secretion in the tracheal, suggesting that naringenin possessed a widely expectorant activity.

MUC5AC is the prime mucin of airway epithelia, which often abnormally expresses and is associated with airflow obstruction and airway hyperresponsiveness in patients with COPD [96,97]. Nie et al. comprehensively investigated the expectorant mechanisms of naringenin in epidermal growth factor (EGF)-induced A549 cells [98]. Naringenin (30 and 100 μM) was found to not only decrease EGF-induced overexpression of MUC5AC but also suppress the phosphorylation of the EGF receptor, p38 mitogen-activated protein kinase (MAPK), extracellular signal-regulated kinase (ERK1/2), c-Jun N-terminal kinase (JNK), NF-κB p65, and activator protein (AP)-1. Yang et al. reported the expectorant activity of naringenin in a human airway epithelial cell model challenged by human neutrophil elastase [99]. Treatment with naringenin (100 μM) significantly downregulated MUC5AC mucin expression, which is associated with the reduction of ROS production and the inhibition of NF-κB activity.

The inhibition of persistent goblet cell differentiation is necessary to reduce intraluminal mucus accumulation, providing a potential way forward in the treatment of chronic airways diseases [100]. Chen et al. proved that naringin could exert mucoactive effects through multiple targets, correlated with the inhibition of goblet cell hyperplasia and mucus hypersecretion, as well as the promotion of sputum excretion in an LPS-induced acute lung injure mice model [101]. The expression of MUC5AC in BALF and goblet cells in large airways was significantly attenuated with naringin (15 and 60 mg/kg, p.o.) treatment. Meanwhile, naringin was found to inhibit the goblet cell hyperplasia in small airways at a high concentration (60 mg/kg). Oral treatment with naringin (12.4 mg/kg) also significantly decreased LPS-induced enhancement of sputum volume and increased the elasticity and viscosity of sputum in the lower trachea of beagle dogs.

Cystic fibrosis transmembrane conductance regulator (CFTR) is a critical airway epithelial Cl- channel that can regulate the electrolytes and fluid secretion across the respiratory system, so a lack of CFTR may lead to the retention of sputum in the airway [102]. Shi et al. demonstrated that naringenin had regulatory effects on the CFTR-mediated Cl^−^ secretion probably through a signaling pathway associated with Na^+^-K^+^-2Cl^−^ co-transporters and K^+^ channels on the basolateral membrane. Furthermore, naringenin (100 μM) could regulate CFTR expression, thereby decreasing the viscosity of sputum in an LPS-induced airway epithelial cell model [103]. In addition, the in vitro and in vivo studies indicated that naringin markedly reduced diesel particulate matter (DPM)-induced liquid viscosity by reducing MUC5AC secretion, increasing CFTR protein expression, and increasing intracellular cAMP to promote CFTR activation [104]. The expectorant effects of naringenin and naringin are summarized in Table 5.

### 2.6. Antitussive

Cough, a source of significant distress of patients, is commonly reported at the time of COPD exacerbation and associated with exacerbation frequency [105]. Although the central antitussives have remained the preferable choice for decades, they have limitations concerning efficacy and safety [106]. Therefore, there is an urgent need to identify an alternative drug to relieve the cough reflex in COPD with lower side effects.

Luo et al. found that naringin (18.4 mg/kg, p.o.) effectively attenuated the airway hyperresponsiveness, thereby attenuating CS exposure enhanced chronic cough in a guinea pig model [107]. However, the precise antitussive mechanisms of naringenin are still not fully understood. Gao et al. reported the antitussive effect and its mechanisms of naringin in different models of experimentally induced cough in guinea pigs [108]. Compared with codeine phosphate (a common central antitussive), naringin (15, 30, and 60 mg/kg, i.v.) did not exert central antitussive effects on cough elicited by electrical stimulation of the superior laryngeal nerve. Meanwhile, naringin (0.5, 1.0, and 2.0 µM) also had no inhibiting effect on the cough reflex induced by stimulation of the trachea after intracerebroventricular injection. These studies suggested that naringin was a peripheral antitussive rather than a central antitussive, which did not exert its antitussive effect through either the sensory neuropeptide system or the modulation of ATP-sensitive K^+^ channels. Smith and Badri suggested that the advantages of peripheral antitussives are the potential avoidance of side effects on the common central nervous system such as drowsiness and the possibility of delivering therapies directly to the airways, thereby reducing the overall risk of systemic adverse events [109].

Airway hyperresponsiveness, one of the major causes of chronic cough, has been considered as a risk factor for the development and progression of COPD [110]. Naringin (18.4 mg/kg, p.o.) was found to significantly alleviate airway hyperresponsiveness, thereby reducing the enhanced cough induced by capsaicin in a cough-variant asthma guinea pig model [111]. In addition, compared with other common peripheral antitussives including levodropropizine and moguisteine, naringin exerted its antitussive effect through remarkably inhibiting the expression of substance P (SP) content and neurokinin (NK)-1 receptor, as well as preventing the decline of neutral endopeptidase (NEP) activity in the lungs [107]. These findings suggest that naringenin might be a promising peripheral antitussive that relieve the suffering of COPD patient. The antitussive effects of naringenin and naringin are summarized in Table 6.

## 3. Network Pharmacology

### 3.1. Data Preparation

Two drug-target databases were used to mine the potential targets of naringenin. Firstly, known targets were obtained from the Traditional Chinese Medicine Systems Pharmacology (TCMSP) database, which is a pharmacology platform of Chinese herbal medicines that includes 499 Chinese herbs registered in the Chinese pharmacopoeia with 29,384 ingredients, 3,311 targets and 837 associated diseases [112]. The second part was derived from Swiss Target Prediction, which can efficiently predict the most probable protein targets of a small molecule [113]. The (sdf) file of naringenin was uploaded into the webtool and filtered by “probability (the probability for a bioactive molecule to have a given protein as target) >0”, with the organism selected as “Homo sapiens”. By merging the two parts of data, we obtained a total of 120 potential protein targets of naringenin.

**Figure 2:**
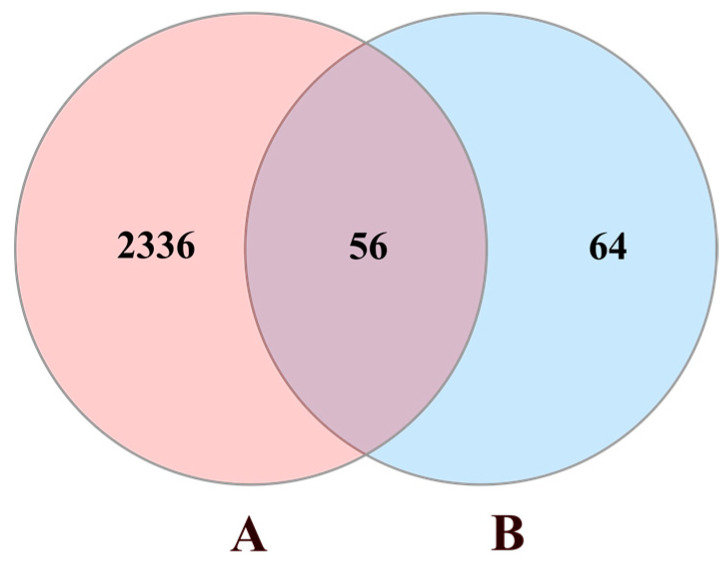
Venn diagram of naringenin-treated Chronic obstructive pulmonary disease (COPD) targets. Circle A represents 2392 COPD-related targets. Circle B represents 120 targets of naringenin. The intersection of two circles represents 56 common targets.

Using “chronic obstructive pulmonary disease” as a keyword, COPD-associated targets were collected from four currently available databases, including the GeneCards, the Online Mendelian Inheritance in Man (OMIM), the Therapeutic Targets Database (TTD), and the DrugBank. After deleting the duplicates, we acquired 2392 COPD-related targets. All the above protein targets were transferred into official gene symbols through the Uniprot database. Targets of naringenin were mapped to the COPD-related targets to obtain 56 common targets (Figure 2).

### 3.2. Protein–Protein Interaction (PPI) Network Construction

**Figure 3:**
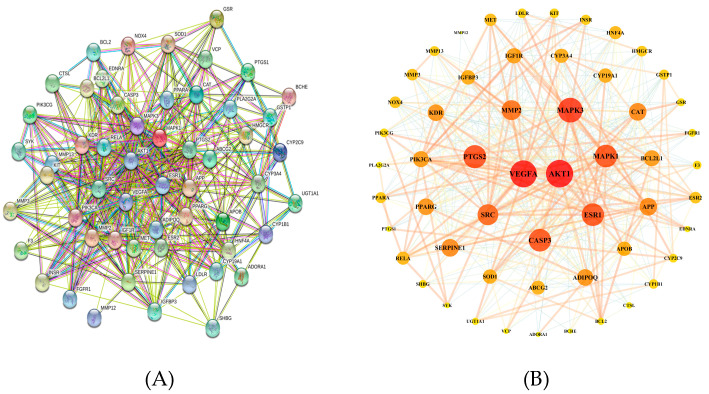
Protein–protein interaction (PPI) network related to naringenin targets-COPD targets (NT-CTs). (**A**) The network in the STRING database. (**B**) The network visualized in Cytoscape software. A high node degree value is represented by a large size and dark color, whereas a low node degree value is represented by a small size and light color.

To further reveal the potential pharmacological effects of naringenin against COPD, we constructed a naringenin targets-COPD targets (NT-CTs) PPI network for these 56 targets in the STRING 11.0 database, with organism species selected as “Homo sapiens” and a confidence score >0.4. The nodes indicate proteins and the edges indicate the interaction between proteins. Hiding a disconnected node, we constructed a network with 55 nodes and 463 edges (Figure 3A). The network was input into Cytoscape 3.6.1 to be visualized. The degree value represents the number of edges connected to the node. As shown in Figure 3B, a high node degree value is represented by a large size and dark color, whereas a low node degree value is represented by a small size and light color. As shown in Table 7, these 55 targets ranking by degree value may be the core targets of naringenin-treated COPD.

### 3.3. GO and KEGG Pathway Enrichment Analysis

**Figure 4:**
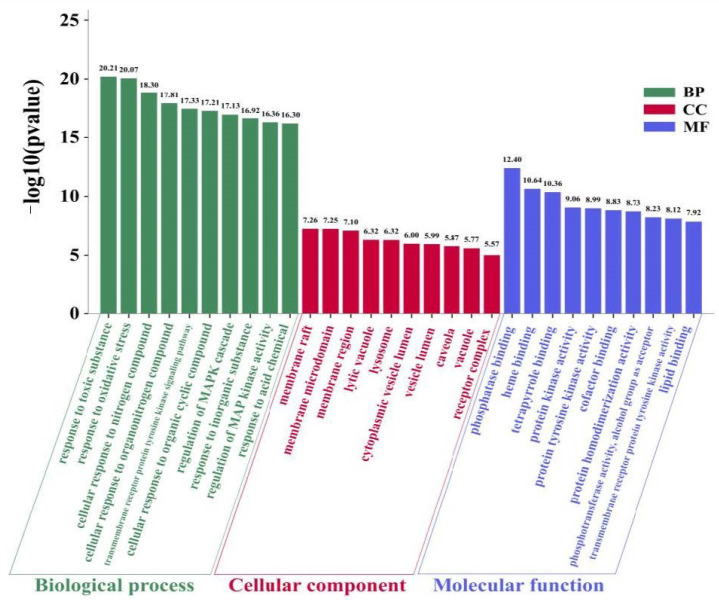
Gene Ontology (GO) enrichment analysis for NT-CTs including three categories: Biological progress (BP), cellular component (CC), and molecular function (MF). The top 10 terms ranking by −log10(*p* value) are shown.

Metascape is a webtool that combines over 40 independent knowledgebases and provides a comprehensive gene list annotation and analysis resource for experimental biologists [114]. Metascape was used to perform Gene Ontology (GO) enrichment analysis of the 56 NT-CTs and the “*p* Value Cutoff <0.05” was set, which included three categories: Biological progress (BP), cellular component (CC), and molecular function (MF). The top 10 significantly enriched terms in the BP, CC, and MF are shown in Figure 4. These results indicate that, in the BP category, NT-CTs are enriched in, e.g., response to toxic substance, response to oxidative stress, cellular response to nitrogen compound, and transmembrane receptor protein tyrosine kinase signaling pathway. In the CC category, these targets are enriched, for example, in membrane raft, membrane microdomain, membrane region, lytic vacuole, and lysosome. In the MF category, these targets are enriched, e.g., in phosphatase binding, heme binding, tetrapyrrole binding, protein kinase activity, and protein tyrosine kinase activity.

**Figure 5:**
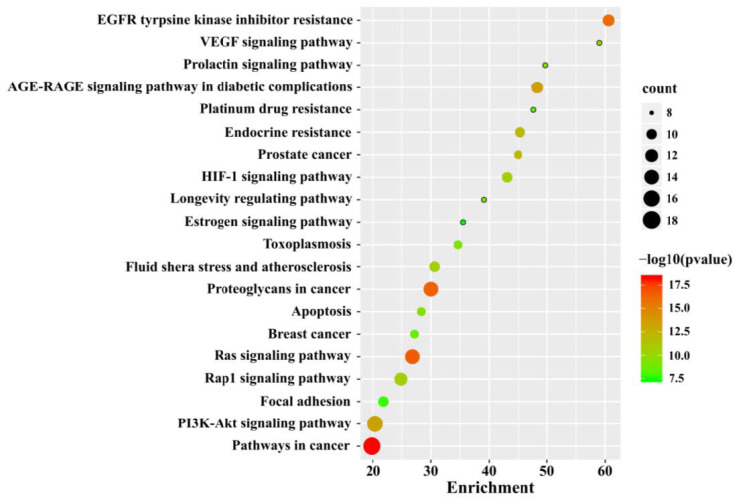
Kyoto Encyclopedia of Genes and Genomes (KEGG) analysis for NT-CTs. The top 20 pathways are shown. The size of the node represents the number of target genes in the pathway and the color of the dot reflects the −log10(*p* value).

We performed Kyoto Encyclopedia of Genes and Genomes (KEGG) signaling pathway enrichment analysis of these NT-CTs based on Metascape. The top 20 significantly enriched pathways are shown in Figure 5. The NT-CTs-based KEGG pathways are mainly enriched in pathways in cancer, proteoglycans in cancer, EGFR tyrosine kinase inhibitor resistance, the PI3K/Akt signaling pathway, the Ras signaling pathway, the AGE-RAGE signaling pathway in diabetic complications, endocrine resistance, the HIF-1 signaling pathway, the Rap1 signaling pathway, and prostate cancer.

Recent studies demonstrated that PI3K signaling is prominently activated in COPD and correlates with increased susceptibility of patients to lung infections [115]. Phosphatase and tensin homolog deleted from chromosome ten (PTEN), a negative regulator of the PI3K pathway, showed lower expression in patients with COPD compared with healthy control and positively correlated with the severity of airflow obstruction [116]. Phosphorylated AKT, as a marker of PI3K activation, was negatively associated with PTEN protein level [117]. In several cell lines, the PTEN level was found to be decreased by cigarette smoke extract (CSE) treatment and thereby activate the PI3K/AKT pathway, resulting in pro-inflammatory cytokine release and macrophage M2 polarization involved in COPD inflammation response [118,119]. The PI3K/AKT pathway also participated in the regulation of airway remodeling, apoptosis, and mucus hypersecretion to accelerate the development of COPD [120,121,122]. Additionally, PI3K inhibitors have been shown to induce alveolar regeneration and restore glucocorticoid function in COPD patients [123,124].

AKT, a wide-range regulatory protein, is collaboratively regulated by multiple upstream proteins and regulates many downstream effectors [125]. Signal transducer and activator of transcription (STAT)3 can activate PTEN and thereby inhibit the PI3K/AKT pathway, which may activate various downstream targets including caspase-3, Bcl-2, VEGF, eNOS, NF-κB, and Nrf2 [115]. The protein levels of Bcl-2 and caspase-3 have been shown to change in CSE-treated cell lines and COPD mice, and these changes are closely related to promoted cell apoptosis [126,127]. eNOS dysfunctionality was aggravated during exacerbations in COPD patients and correlates with airway inflammatory markers [128]. The variants and combinations of polymorphisms of eNOS likely contributed to oxidative stress in COPD [129]. There is ample evidence that NF-κB and Nrf2 pathways were participants in the regulation of a broad spectrum of inflammatory and oxidative stress networks in COPD [130,131].

### 3.4. Analysis of miRNA-Mediated Naringenin in the Treatment of COPD

MicroRNAs (miRNAs) have been implicated in the development of COPD through the transcriptional and translational modulation of important genes, so it is necessary to analyze the potential role of the miRNA-mediated treatment of COPD with naringenin [132]. Using the PubMed database, eight miRNAs regulated by naringenin including miR-29b-3p, miR-29c-3p, miR-17-3p, miR-25-5p, miR-223-3p, let-7a, miR-224-3p, and miR-140-3p were collected through a literature search. Naringenin was found to exert antioxidant activity and neuroprotective effect in vitro by increasing the level of miR-17-3p and decreasing the expression of miR-224-3p respectively [133,134]. Liang et al. revealed that naringenin suppressed the activation of Smad3 and upregulated the expression of miR-29b-3p and miR-29c-3p, thereby inhibiting fibrosis in cardiac fibroblasts [135]. In addition, naringenin inhibited spinal cord injury-induced activation of neutrophils by repressing the level of miR-223 in rats [136]. Meanwhile, Yan et al. found that naringenin ameliorated kidney injure by inhibiting the activation of TGF-β1/smads signaling by upregulating let-7a in diabetic nephropathy rats [137]. Defective insulin receptor signaling in patients with gestational diabetes was related to the overexpression of miR-140-3p and naringenin was found to downregulate the level of miR-140-3p to protect trophoblasts and endothelial cells from the harm of a high glucose environment [138]. Nevertheless, naringenin interacts with these miRNAs at an atomic level has not been well investigated, which needs further research.

**Figure 6:**
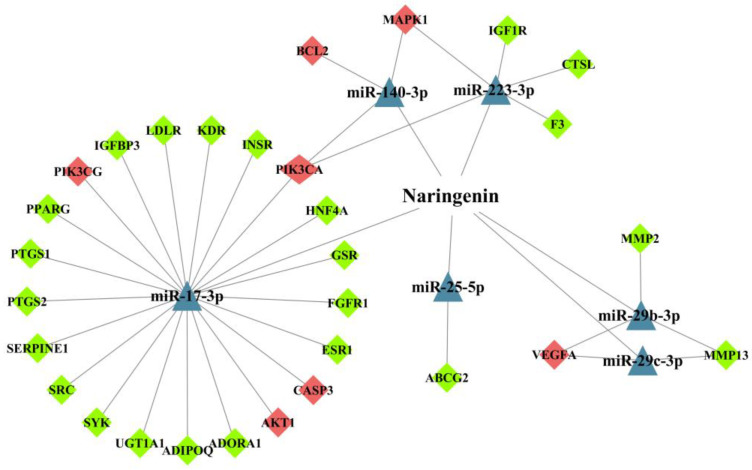
The network for microRNAs (miRNAs)-mediated targets of naringenin in the treatment of COPD. The triangles represent miRNAs and diamonds represent target genes. Red diamonds represent targets that may be relevant to COPD pathogenesis.

The target genes of these miRNAs were predicted using the Targetscan database and the miRDB database. These predicted genes were intersected with NT-CTs to obtain the potential miRNA-mediated targets of naringenin in the treatment of COPD. Hiding the let-7a and miR-224-3p without connected targets, we visualized the network for miRNA-mediated targets of naringenin to further explore its potential therapeutic mechanisms in COPD. As shown in Figure 6, triangles represent miRNAs, and diamonds represent targets. The red targets are relatively important targets, which are probably associated with the pathogenesis of COPD. PIK3CA and PIK3CG are genes that encode the p110 catalytic subunit which is a necessary component of PI3K. In the miRNA-mediated network, PIK3CA connects with three miRNAs including miR-17-3p, miR-140-3p, and miR-223-3p, and PIK3CG and AKT1 connect with miR-17-3p. Other possible targets associated with COPD pathogenesis, such as BCL2 and CASP3 are also linked with corresponding miRNAs. These findings may provide a perspective complement to the underlying mechanisms of naringenin in the treatment of COPD.

It is worth mentioning that vascular endothelial growth factor A (VEGFA) possesses the second highest degree value in the PPI network and connects with miR-29 family members in the miRNAs network. A clinical study reported that genetic polymorphisms of VEGF, the most important candidate angiogenic factor, were associated with the progression of COPD [139]. The PI3K/AKT pathway directly or via the eNOS signaling pathway results in the overexpression of VEFG and participate in the process of angiogenesis involved in multiple lung disorders [140]. These findings suggested that naringenin may affect the process of angiogenesis in COPD by targeting VEGF through the mediation of specific miRNAs.

## 4. Conclusions

COPD is a heterogeneous and complex disease characterized by persistent inflammation in the respiratory system involving multiple signaling pathways [141]. Though many previous studies have demonstrated the clinical potential of naringenin in treating COPD by both preventive and therapeutic measures, they are scattered and unsystematic. Through network pharmacology analysis, we systematically integrate available potential targets and pathways that treat COPD by naringenin and further predict new targets and pathways to construct a prospective regulatory network.

**Figure 7:**
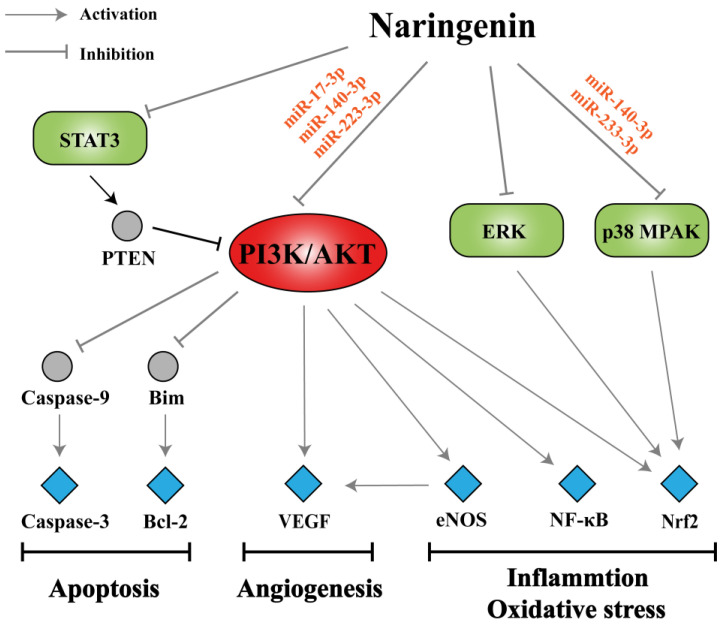
Summary of potential mechanisms of naringenin in the treatment of COPD. Naringenin has been shown to act on various signaling pathways such as phosphatidylinositol 3-kinase (PI3K)/protein kinase B (AKT), STAT3, p38 mitogen-activated protein kinase (MAPK), and extracellular signal-regulated kinase (ERK) pathways probably through specific miRNAs. The PI3K/AKT pathway, a bridge in the regulatory network, can activate downstream effectors including caspase-3, Bcl-2, VEGF, eNOS, NF-κB, and Nrf2 thereby participating in the processes of apoptosis, angiogenesis, inflammation, and oxidative stress in COPD pathogenesis.

As mentioned in this literature review, naringenin has been shown to exert potential pharmacological activities against multiple pathological stages of COPD through various signaling pathways such as PI3K/AKT, STAT3, p38 MAPK, and ERK pathways. Based on network pharmacology, we consider that miRNAs may act as upstream regulators on corresponding signaling pathways, and the PI3K/AKT signaling pathway acts as a bridge in the regulatory network of naringenin in the treatment of COPD. The PI3K/AKT pathway can activate downstream effectors including caspase-3, Bcl-2, VEGF, eNOS, NF-κB, and Nrf2, thereby participating in the processes of apoptosis, angiogenesis, inflammation, and oxidative stress in COPD pathogenesis (Figure 7). As a possible PI3K inhibitor, naringenin is expected to be applied in COPD treatment. However, the therapeutic effects of naringenin through PI3K pathway mediation have not been well studied in COPD models. Angiogenesis partakes in the remodeling of airways in COPD, probably as part of the inflammatory response to smoking, but its specific role in disease progression has not been fully elucidated [142]. Bakakos et al. suggested that advances in understanding the role of angiogenesis in COPD might identify new therapeutic targets that could affect the natural history of the disease [143]. In addition, there are no reports on the regulation of apoptosis in COPD with naringenin treatment, which is noteworthy.

Due to the complexity, heterogeneity, and different severity of COPD, specific clinical stages and phenotypes of COPD for which naringenin is most appropriate remains to be further explored. Current studies are typically carried out with animal or cell line models, thus more clinical trials are needed to further support the use of naringenin in humans. Clinal application of naringenin is limited by its poor aqueous solubility and bioavailability in humans, therefore, it is necessary to develop better drug delivery systems to be used by patients [11]. Recently, delivering naringenin as an aerosol via pulmonary route allows rapid absorption and high local concentration, which might be a feasible administration route in the treatment of COPD [144]. In addition, dosage regimen, safety, and efficacy of naringenin should be identified in COPD before applicating in humans. With a clearer understanding of the underlying mechanisms of COPD with naringenin treatment, this flavanone might be a promising tactic of clinical treatment for COPD in the near future.

## Figures and Tables

**Table 1:** Summary of anti-inflammatory activities of naringenin and naringin.

Pharmacological Activity	Type of Study	Study Subject	Pharmacological Aspects	Findings	Ref.
**Anti-inflammation**	In vivo	LPS-induced acute lung injury mice	Naringin; 15, 30, and 60 mg/kg, p.o.	Pulmonary neutrophil infiltration and TNF-α, MPO, iNOS, and NF-κB activities ↓	[23]
	In vivo	CS-exposed rats	Naringin; 20, 40, and 80 mg/kg, p.o.	Infiltration of neutrophils and MPO, MMP-9, TNF-α, and IL-8 levels ↓; Level of IL-10 ↑	[24]
	In vivo	*Staphylococcus aureus*-induced pneumonia mice	Naringenin; 100 mg/kg, i.h.	Pulmonary inflammation and inflammatory cells infiltration ↓	[25]
	Both in vitro and in vivo	LPS-induced RAW 264.7 cell line; CLP-induced mice	Naringin; 50, 100, 200 μM (in vitro) 200 mg/kg, i.p. (in vivo)	TNF-α expression and HMGB1 release ↓; HO-1 expression via the AMPK-p38-Nrf2 pathway ↓ (in vitro) Lung injury ↓; TNF-α and HMGB1 expression ↓ (in vivo)	[26]
	Both in vitro and in vivo	CS-exposed A549 cell line and mice	Naringenin; 2, 20, 50 mM (in vitro) 20, 40, and 80 mg/kg, p.o. (in vivo)	NF-κB activity ↓; Levels of GR mRNA and protein ↑ (in vitro) Inflammatory cells and the production of IL-8, TNF-α, and MMP-9 ↓ (in vivo)	[33]
	In vivo	CS-exposed chronic bronchitis guinea pigs	Naringin; 9.2, 18.4 and 36.8 mg/kg, p.o.	Levels of IL-8 and TNF-α and MPO ↓	[34]
	In vivo	LPS-induced acute lung injury mice	Naringenin; 100 mg/kg, p.o.	Pulmonary edema, neutrophil infiltration and the levels of TNF-α, IL-1β, IL-6, and MIP-2 ↓; The activities of PI3K and AKT ↓	[36]
	In vivo	Radiation-induced lung injury mice	Naringenin; 100 and 200 mg/kg, p.o.	Level of IL-1β ↓	[37]
	In vivo	LPS-induced acute lung injury rats	Naringenin; 50 and 100 mg/kg, p.o.	Levels of IL-6, MPO, TNF-α, and caspase-3 ↓; HSP70 expression ↑	[12]
	In vivo	Carrageenan-induced pleurisy mice	Naringin; 40 and 80 mg/kg, p.o.	Th1 cytokines (TNF-α, IL-2, IL-6, and IL-17) ↓; NF-κB and STAT3 activities↓; Th2 cytokines (IL-4 and IL-10) ↑	[46]
	In vitro	LPS-induced RAW 264.7 cell line	Naringin; 50, 100, and 200 μM	Secretion of IL-8, MCP-1 and MIP-1α ↓; NF-κB and MAPK activities ↓	[49]
	In vivo	Allergen-induced asthma mice	Naringenin; 25, 50, and 100 mg/kg, i.p.	Levels of CCL5 and CCL11 and NF-κB activity ↓	[50]
	In vitro	LPS-induced acute lung injury mice	Naringenin; 100 μM	TSLP production and levels of RIP-2 and caspase-1 ↓	[53]
	In vitro	LPS-induced RAW 264.7 cell line	Naringenin NPs; 25μg/mL	NF-κB and MAPK activities ↓; Levels of TNF-α, IL-6, MCP-1, and IL-1β ↓	[55]

**Table 2:** Summary of antioxidative activities of naringenin and naringin.

Pharmacological Activity	Type of Study	Study Subject	Pharmacological Aspects	Findings	Ref.
**Antioxidation**	In vivo	LPS-induced acute lung injury mice	Naringenin; 100 mg/kg, p.o.	Levels of H_2_O_2_ and MDA ↓	[36]
	In vivo	Benzo[a]pyrene-induced rats	Naringenin; 100 mg/kg, p.o.	Levels of GSH, GPx, GST, GR, SOD, CAT, and XO ↑; Expression of COX-2 through blockage of NF-κB ↓	[66]
	In vitro	Paraquat-induced BEAS-2B cell line	Naringenin; 100 μM	Generation of ROS ↓; Antioxidant-related genes including GPX2, GPX3, GPX5, and GPX7 and Nrf2 activity ↑	[67]
	In vivo	Wood smoke-exposed rats	Naringin; 80 mg/kg, p.o.	The activities of SOD and CAT ↑; Levels of NO ↓	[70]
	In vivo	Monocrotaline-induced pulmonary hypertension rats	Naringenin; 50 mg/kg, p.o.	GSH content and eNOS protein expression ↑; Expression of iNOS ↓	[71]
	In vitro	LPS-induced RAW 264.7 cell line	Naringenin NPs; 25 μg/mL	Expression of iNOS and COX-2 and the production of NO ↓	[55]

**Table 3:** Summary of anti-airway remodeling activities of naringenin and naringin.

Pharmacological Activity	Type of Study	Study Subject	Pharmacological Aspects	Findings	Ref.
**Anti-Airway Remodeling**	In vivo	House dust mite-induced asthma mice	Naringenin; 9 mg/mL, p.o.	Subepithelial fibrosis and smooth muscle hypertrophy ↓	[76]
	In vivo	Ovalbumin-induced asthma mice	Naringin; 5 and 10 mg/kg, p.o.	Mean airway resistance and the level of IgE ↓ Percentage of Th1/Th2 cells ↑	[80]
	In vivo	Ovalbumin-induced asthma mice	Naringenin; 50 mg/kg, i.p.	Area of airway fibrosis and the levels of Th2 cytokines ↓	[81]
	In vivo	CS-exposed rats	Naringin; 20, 40, and 80 mg/kg, p.o.	Thickening of the bronchial wall ↓	[24]

**Table 4:** Summary of anti-pulmonary fibrosis activities of naringenin and naringin.

Pharmacological Activity	Type of Study	Study Subject	Pharmacological Aspects	Findings	Ref.
**Anti-Pulmonary Fibrosis**	In vivo	Paraquat-induced pulmonary fibrosis mice	Naringin; 60 and 120 mg/kg, p.o.	Expression of TNF-α, MMP-9, and TIMP-1 and the pulmonary fibrosis deposition ↓	[86]
	In vivo	Bleomycin-induced fibrosis rats	80 mg/kg, p.o.	Levels of HYP and lung collagen content ↓	[88]
	Both in virto and in vivo	*Mycoplasma pneumoniae*-induced BEAS-2B cell line and pneumonia mice	Naringenin; 100 μM (in vitro) 100 mg/kg, p.o. (in vivo)	Fibrosis-related proteins (TGF-β, α-SMA, collagen I and collagen III) expression and autophagy ↓ (in vitro) Level of TGF-β and autophagy relative protein LC3 and Beclin-1 expression ↓ (in vivo)	[90]

**Table 5:** Summary of expectorant effects of naringenin and naringin.

Pharmacological Activity	Type of Study	Study Subject	Pharmacological Aspects	Findings	Ref.
**Expectorant**	In vivo	Several animal models	Naringenin; 30–67 mg/kg, p.o.	Volume of airway secretions ↑ (mice); Mucociliary clearability and tracheal mucociliary velocity ↑ (pigeons); Mucin secretion ↓ (rats)	[95]
	In vitro	EGF-induced A549 cell line	Naringenin; 30 and 100 μM	Expression of MUC5AC and phosphorylation of EGF receptor, MAPK, ERK1/2, JNK, NF-κB p65, and AP1 ↓	[98]
	In vitro	Human neutrophil elastase induced-human airway epithelial cell line	Naringenin; 100 μM	MUC5AC expression, production of ROS and NF-κB activity ↓	[99]
	In vivo	LPS-induced mice and beagle dogs	Naringin; 15 and 60 mg/kg, p.o. (mice); 12.4 mg/kg, p.o. (beagle dogs)	Expression of MUC5AC and goblet cell hyperplasia ↓ (mice); Sputum volume ↓ and elasticity and viscosity of sputum ↑ (beagle dogs)	[102]
	In vitro	LPS-induced airway epithelial cell and Calu-3 cell line	Naringenin; 100 μM	CFTR expression ↑ by Na^+^-K^+^-2Cl^−^ co-transporters and K^+^ channels and regulated by intracellular cAMP	[103]
	Both in vitro and in vivo	DPM-induced Calu-3 cell line and mice	Naringenin; 25, 50, 100 μM (in vitro); Naringin; 30, 60, and 120 mg/kg, p.o. (in vivo)	Liquid viscosity, MUC5AC and total protein secretion ↓; CFTR, AQP1, and AQP5 expression and intracellular cAMP ↑	[104]

**Table 6:** Summary of antitussive effects of naringenin and naringin.

Pharmacological Activity	Type of Study	Study Subject	Pharmacological Aspects	Findings	Ref.
**Antitussive**	In vivo	CS-exposed guinea pigs	Naringin; 18.4 mg/kg, p.o.	Airway hyperresponsiveness, chronic cough and expression of SP content, NK-1 receptor and NEP activity ↓	[107]
	In vivo	Different cough guinea pig models	Naringin; 15, 30, and 60 mg/kg, i.v. 0.5, 1.0, and 2.0 µM, i.c.v.	Exerted peripheral antitussive effects	[108]
	In vivo	Capsaicin-induced cough-variant asthma guinea pigs	Naringin; 18.4 mg/kg, p.o.	Airway hyperresponsiveness and cough ↓	[111]

**Table 7:** The list of 55 core targets ranked by degree value.

NO	Gene Name	Protein Name	Degree	NO	Gene Name	Protein Name	Degree
1	*AKT1*	RAC-alpha serine/threonine-protein kinase	42	29	*MMP3*	Stromelysin-1	14
2	*VEGFA*	Vascular endothelial growth factor A	41	30	*NOX4*	NADPH oxidase 4	14
3	*MAPK3*	Mitogen-activated protein kinase 3	37	31	*PPARA*	Peroxisome proliferator-activated receptor alpha	13
4	*PTGS2*	Prostaglandin G/H synthase 2	34	32	*HMGCR*	3-hydroxy-3-methylglutaryl-coenzyme A reductase	13
5	*ESR1*	Estrogen receptor	33	33	*INSR*	Insulin receptor	12
6	*MAPK1*	Mitogen-activated protein kinase 1	33	34	*MMP13*	Collagenase 3	12
7	*CASP3*	Caspase-3	33	35	*GSTP1*	Glutathione S transferase P	12
8	*SRC*	Proto-oncogene tyrosine-protein kinase Src	30	36	*LDLR*	Low-density lipoprotein receptor	11
9	*MMP2*	72 kDa type IV collagenase	28	37	*KIT*	Mast/stem cell growth factor receptor Kit	11
10	*CAT*	Catalase	24	38	*CYP1B1*	Cytochrome P450 1B1	11
11	*SERPINE1*	Plasminogen activator inhibitor 1	24	39	*PIK3CG*	Phosphatidylinositol 4,5-bisphosphate 3-kinase catalytic subunit gamma isoform	10
12	*APP*	Amyloid-beta precursor protein	24	40	*CYP2C9*	Cytochrome P450 2C9	10
13	*KDR*	Vascular endothelial growth factor receptor 2	22	41	*F3*	Tissue factor	10
14	*ADIPOQ*	Adiponectin	22	42	*GSR*	Glutathione reductase, mitochondrial	9
15	*PPARG*	Peroxisome proliferator-activated receptor gamma	22	43	*FGFR1*	Fibroblast growth factor receptor 1	9
16	*PIK3CA*	Phosphatidylinositol 4,5-bisphosphate 3-kinase catalytic subunit alpha isoform	21	44	*SHBG*	Sex hormone-binding globulin	9
17	*BCL2L1*	Bcl-2-like protein 1	21	45	*BCL2*	Apoptosis regulator Bcl-2	8
18	*IGF1R*	Insulin-like growth factor 1 receptor	20	46	*UGT1A1*	UDP-glucuronosyltransferase 1A1	8
19	*SOD1*	Superoxide dismutase	18	47	*EDNRA*	Endothelin-1 receptor	7
20	*APOB*	Apolipoprotein B-100	18	48	*PLA2G2A*	Phospholipase A2	7
21	*CYP3A4*	Cytochrome P450 3A4	18	49	*CTSL*	Procathepsin L	7
22	*IGFBP3*	Insulin-like growth factor-binding protein 3	18	50	*SYK*	Tyrosine-protein kinase SYK	6
23	*CYP19A1*	Aromatase	18	51	*VCP*	Transitional endoplasmic reticulum ATPase	6
24	*ABCG2*	Broad substrate specificity ATP-binding cassette transporter ABCG2	17	52	*PTGS1*	Prostaglandin G/H synthase 1	6
25	*RELA*	Transcription factor p65	16	53	*ADORA1*	Adenosine receptor A1	5
26	*HNF4A*	Hepatocyte nuclear factor 4-alpha	16	54	*BCHE*	Cholinesterase	5
27	*MET*	Hepatocyte growth factor receptor	15	55	*MMP12*	Macrophage metalloelastase	2
28	*ESR2*	Estrogen receptor beta	14				

## Abbreviations

COPD	chronic obstructive pulmonary disease
CS	cigarette smoke
LPS	lipopolysaccharide
CLP	cecum ligation and puncture
MMP	matrix metalloproteinase
BALF	bronchoalveolar lavage fluid
ECM	extracellular matrix
PI3K	phosphatidylinositol 3-kinase
AKT	protein kinase B
MCP-1	monocyte chemoattractant protein-1
MIP-1α	macrophage inflammatory protein-1α
TSLP	thymic stromal lymphopoietin
RIP-2	receptor-interacting protein-2
PVP	polyvinyl pyrrolidone
NPs	nanoparticles
MAPK	P38 mitogen-activated protein kinase
ROS	reactive oxygen species
MDA	malondialdehyde
SOD	superoxide dismutases
CAT	catalases
XO	xanthine oxidase
GPx	glutathione peroxidases
GSH	glutathione
GST	glutathione s-transferase
GR	glutathione reductase
COX-2	cyclooxygenase-2
iNOS	inducible nitric oxide synthase
eNOS	endothelial nitric oxide synthase
TIMP-1	tissue inhibitor of metalloproteinase-1
HYP	hydroxyproline
CFTR	cystic fibrosis transmembrane conductance regulator
SP	substance P
NK-1	neurokinin-1
NEP	neutral endopeptidase
PPI	protein-protein interaction
NT-CTs	naringenin targets-COPD targets
GO	Gene Ontology
BP	biological progress
CC	cellular component
MF	molecular function
KEGG	Kyoto Encyclopedia of Genes and Genomes
PTEN	phosphatase and tensin homolog deleted from chromosome ten
VEGF	vascular endothelial growth factor
